# Mortality in septic patients treated with vitamin C: a systematic meta-analysis

**DOI:** 10.1186/s13054-020-03438-9

**Published:** 2021-01-06

**Authors:** Sean S. Scholz, Rainer Borgstedt, Nicole Ebeling, Leoni C. Menzel, Gerrit Jansen, Sebastian Rehberg

**Affiliations:** 1 Department of Anaesthesiology, Intensive Care, Emergency Medicine, Transfusion Medicine and Pain Therapy, Protestant Hospital of the Bethel Foundation, University Hospital OWL, University of Bielefeld, Campus Bielefeld-Bethel, Burgsteig 13, Haus Gilead I, 33617 Bielefeld, Germany; 2 Institute for Diagnostic and Interventional Radiology, Protestant Hospital of the Bethel Foundation, Bielefeld, Germany

**Keywords:** Ascorbic acid, Septic shock, Mortality, Sepsis

## Abstract

**Background:**

Supplementation of vitamin C in septic patients remains controversial despite eight large clinical trials published only in 2020. We aimed to evaluate the evidence on potential effects of vitamin C treatment on mortality in adult septic patients.

**Methods:**

Data search included PubMed, Web of Science, and the Cochrane Library. A meta-analysis of eligible peer-reviewed studies was performed in accordance with the PRISMA statement. Only studies with valid classifications of sepsis and intravenous vitamin C treatment (alone or combined with hydrocortisone/thiamine) were included.

**Results:**

A total of 17 studies including 3133 patients fulfilled the predefined criteria and were analyzed. Pooled analysis indicated no mortality reduction in patients treated with vitamin C when compared to reference (risk difference − 0.05 [95% CI − 0.11 to − 0.01]; *p* = 0.08; *p* for Cochran *Q* = 0.002; *I*^2^ = 56%). Notably, subgroup analyses revealed an improved survival, if vitamin C treatment was applied for 3–4 days (risk difference, − 0.10 [95% CI − 0.19 to − 0.02]; *p* = 0.02) when compared to patients treated for 1–2 or > 5 days. Also, timing of the pooled mortality assessment indicated a reduction concerning short-term mortality (< 30 days; risk difference, − 0.08 [95% CI − 0.15 to − 0.01]; *p* = 0.02; *p* for Cochran *Q* = 0.02; *I*^2^ = 63%). Presence of statistical heterogeneity was noted with no sign of significant publication bias.

**Conclusion:**

Although vitamin C administration did not reduce pooled mortality, patients may profit if vitamin C is administered over 3 to 4 days. Consequently, further research is needed to identify patient subgroups that might benefit from intravenous supplementation of vitamin C.

## Background

Sepsis is a life-threatening condition affecting annually more than 48 million patients worldwide. This leads to more than 10 million deaths every year representing the cause of nearly 20% of all global deaths [1]. A cornerstone of its pathophysiology is based on reactive oxygen species (ROS)-driven modification of proteins affecting cellular signaling, gene expression, and other essential cellular processes which are initiated by enzymes such as nicotinamide adenine dinucleotide phosphate oxidase, uncoupling of mitochondrial oxidative phosphorylation, and endothelial nitric oxide synthase [2–5].

Vitamin C mitigates apoptosis by preserving the integrity of the endothelial barrier and counteracts these enzymes that propagate ischemia–reperfusion injury [3]. This was demonstrated in vitro with cultured endothelial cells where nicotinamide adenine dinucleotide phosphate oxidase is the major source of ROS [3]. Furthermore, vitamin C has been proven to play a crucial role in the microcirculation and organ function in animal models and volunteer studies [3, 6–8].

Consequently, intravenous supplementation of vitamin C was investigated in multiple clinical studies exploring the effects in septic patients [8–25]. Early studies such as the randomized, double-blind, placebo-controlled phase I safety trial published by Fowler et al. and the retrospective before–after study by Marik et al. demonstrated its safety and suggested reduced mortality rates [8, 19]. However, following studies provided contradicting results with respect to the effects of vitamin C on mortality prompting uncertainty in the community [8–25].

As a consequence, potential beneficial or detrimental effects could not be sustainably determined by previously published meta-analyses due to divergent patient subsets, heterogenous interventions, and limited numbers of studies included [26–28]. Notably, only in 2020, eight new large clinical trials were published supporting the high interest and relevance of this topic [13, 14, 16, 20–22, 24, 25]. However, consistent data on the effects on mortality of septic patients treated with intravenous vitamin C are still lacking. Therefore, we performed the present meta-analysis on mortality of septic patients treated with vitamin C alone or combined with hydrocortisone/thiamine when compared to standard care.

## Methods

This systematic review and meta-analysis was performed based on a predefined protocol, registered at the international PROSPERO database for prospective systematic reviews (CRD42020185080), and was carried out in accordance with the PRISMA Guidelines [29].

### Study protocol

A systematic literature search was completed for all peer-reviewed and published studies reporting the effects of intravenous vitamin C treatment alone or in combination with thiamine and/or hydrocortisone, when compared to standard care or placebo treatment. Patient population consisted of adult (≥ 18 years) septic patients. Studies had to provide valid data on the mortality rates as well as on timing of mortality assessments. Otherwise, they were excluded. Besides mortality, duration of vasopressors and length of ICU treatment were collected. Regarding missing data, corresponding authors were contacted. Additionally, only original manuscripts published in English were included. There were no restrictions regarding the number of included patients or minimal dosing of vitamin C.

### Literature research and data extraction

**Fig. 1:**
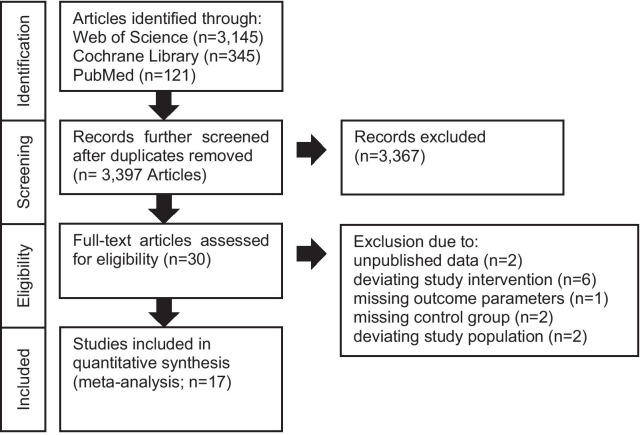
PRISMA flow diagram showing search and selection strategies

Two investigators (S.S.S./G.J.) searched PubMed, Web of Science, Clinical.Trials.gov, and the Cochrane Library independently for eligible studies published until August 2020 (Query Date: August 30, 2020). The search was performed using the terms: (vitami* C OR ascorbic acid) and (sepsi* OR septic OR critic*). Web of Science was searched using topic and articles, whereas PubMed was searched for the category’s clinical and randomized controlled trials. Furthermore, we searched already published meta-analyses and screened the included studies and references [27, 28, 30–32]. Detailed search strategy is visualized in Fig. 1. The same investigators screened the search results according to the title and abstract, reviewed the full-text articles, considered the study for inclusion, and extracted appropriate data from the publications [8–25, 33–45].

### Assessment of bias

Bias within and across the studies was assessed based on the ROBINS-I criteria in non-randomized studies by the Cochrane Bias Methods Group and using the Jadad score for randomized studies [46, 47]. In case of disagreements between the two investigators, a third investigator was consulted.

### Statistical analysis

The effects of the intervention on mortality were investigated by assessing the risk difference in-between the vitamin C and control group by pooling the available data on mortality regarding the longest observational period for each study. Sensitivity analysis included treatment of vitamin C only versus a combination of vitamin C, thiamine, and hydrocortisone. In addition, analyses of all available measurements within the studies (some studies provided multiple measurements) were performed. Subgroup analyses were separated prior analysis and further assessed different measurement periods regarding the pooled analysis as well as the available data on mortality. Furthermore, average patients age was used for subgroup analysis, as well as the duration of vitamin C treatment 1–2 days, 3–4 days, and ≥ 5 days. Risk differences and pooled risk differences were determined and presented using Forrest plots along with respective 95% confidence intervals. A fixed- or random-effects model (Mantel–Haenszel) was used to pool the data, where appropriate. Statistical heterogeneity between the trials was evaluated using Cochran’s *Q* test and *I*^2^ statistic as a measure of variability. Relevant statistical heterogeneity was considered as Cochran’s *Q*-test *p* < 0.05 and *I*^2^ > 50%, in which case a random-effects model was used to estimate the results. Potential publication bias for the specific outcome was explored visually with Funnel plots. The standard error of each trial was plotted against the risk difference using Review Manager (RevMan). Asymmetry in the Funnel plot was considered as presence of publication bias. If within one study more than one measurement was existing, the measurement with the longest observational period was used for the meta-analysis. Values are expressed as mean ± standard deviation (SD). The statistical analyses were performed using RevMan version 5.4 (2020, The Cochrane Collaboration). A two-sided *p* value ≤ 0.05 was considered as statistically significant.

## Results

**Fig. 2:**
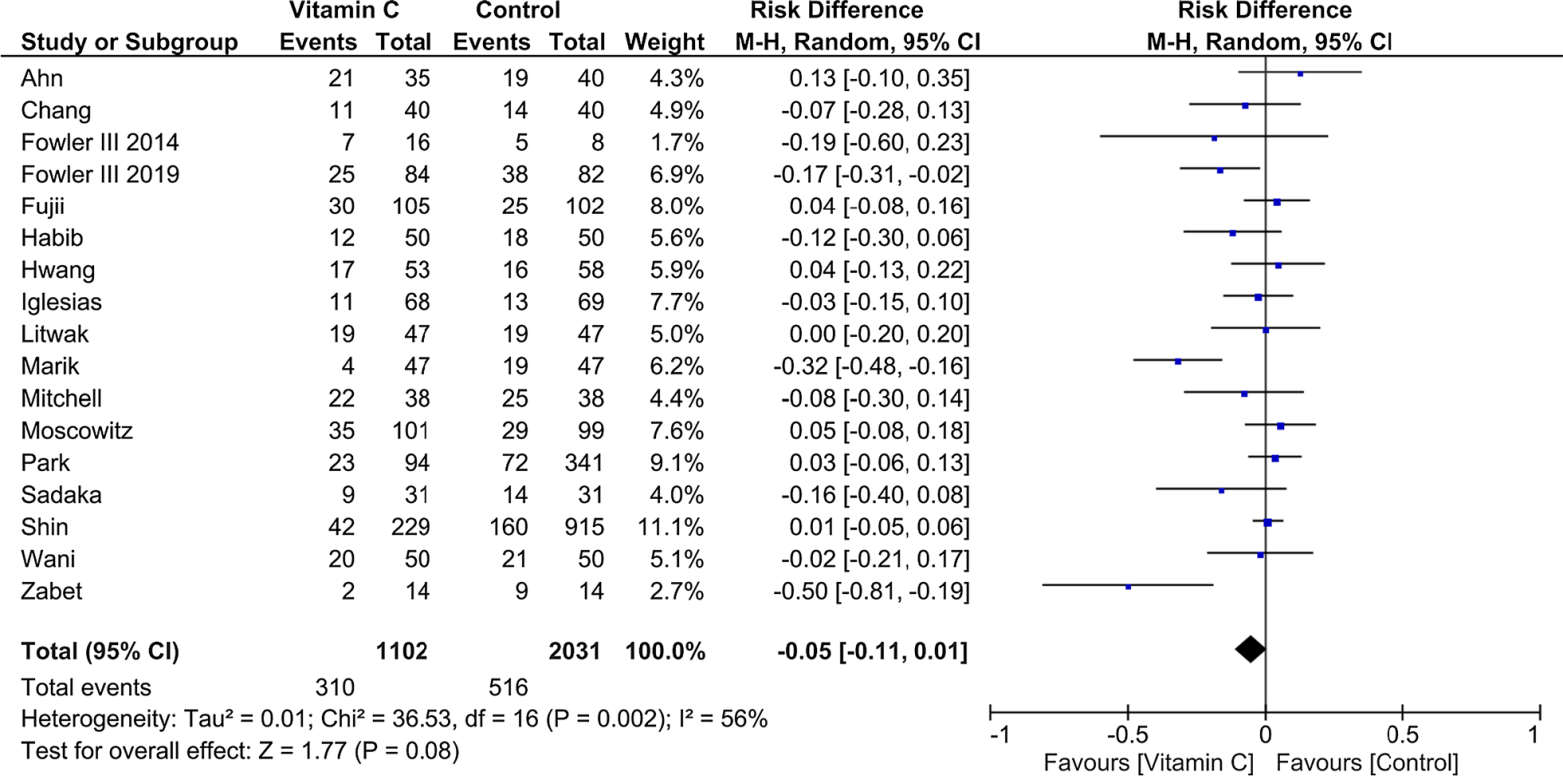
Pooled mortality regarding the longest available time period within each study, risk difference, vitamin C treatment versus control; *M-H* Mantel–Haenszel, *CI* confidence interval

**Fig. 3:**
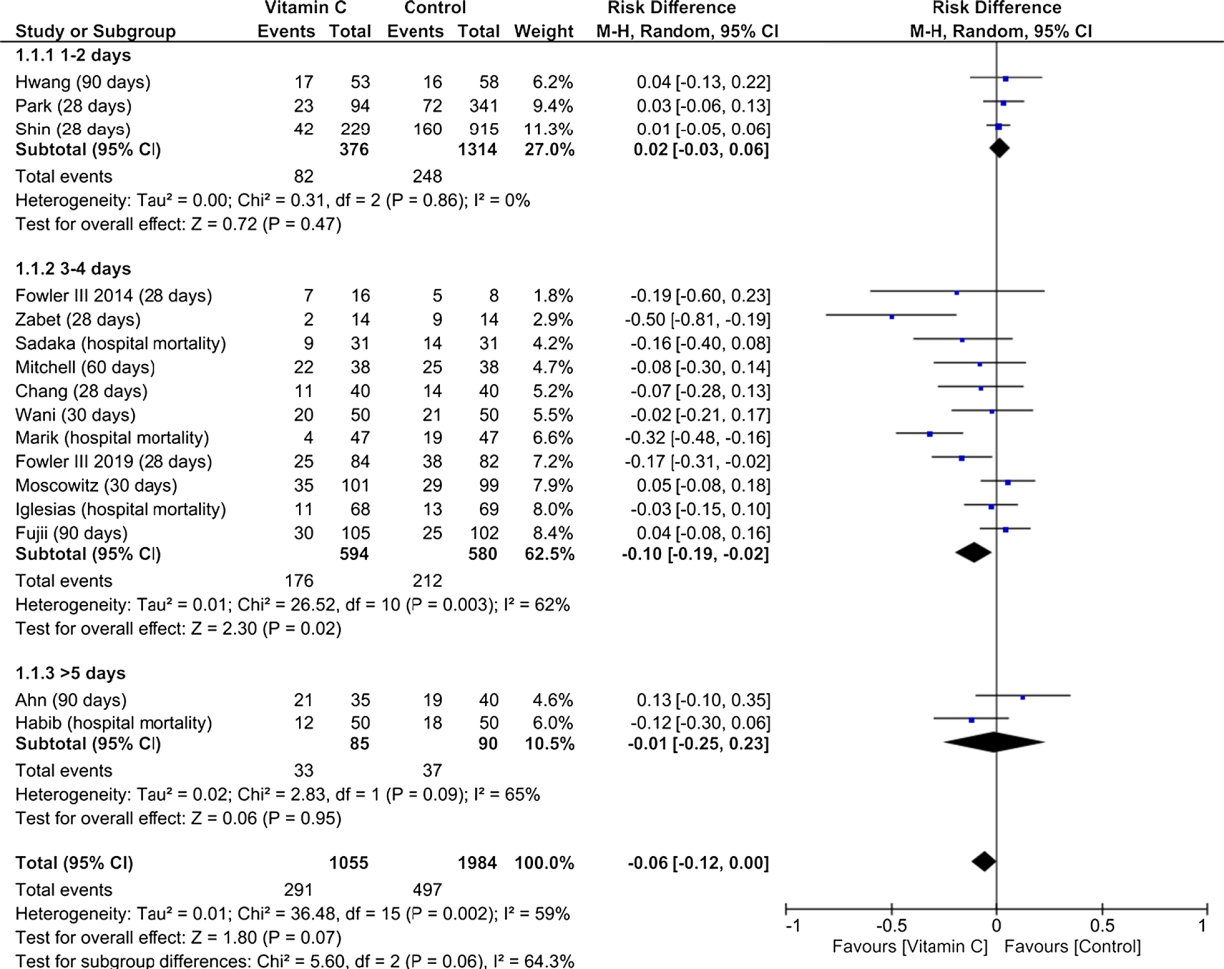
Pooled mortality regarding the longest available time period within each study including subgroup analysis on treatment duration, risk difference, vitamin C treatment versus Control; *M-H* Mantel–Haenszel, *CI* confidence interval

The initial literature search identified 3611 studies from various databases. After duplicates were removed (*n* = 214), 30 articles were identified as potentially appropriate. Following full-text review, a total of 13 studies were excluded due to unpublished data (*n* = 2) [33, 34], deviating study interventions (*n* = 6) [35–40], missing outcome parameters (*n* = 1) [41], missing control group, (*n* = 2) [42, 43], and deviating study populations (*n* = 2) [44, 45]. The included 17 trials summarized randomized and non-randomized, blinded and unblinded, prospective and retrospective, and single- and multi-center studies (Table 1 and Additional file 1: Table S1). Common inclusion criteria were age ≥ 18 years and sepsis or septic shock, based on eligible classifications (Table 1). Common exclusion criteria were limitation of therapy (do-not-resuscitate or intubate orders), imminent death, contraindication for any of the study drugs, and pregnant or lactating women. Interventions were relatively homogenous with a dosing of 1.5 g of vitamin C every 6 h, 100 mg thiamine every 6 h, and 50 mg hydrocortisone every 6 h. However, initiation and duration of the intervention differed considerably within the studies (Additional file 1: Table S2). Patient characteristics were representative of hospitalized septic patients and relatively homogeneous across all studies (Table 1). A lower average patient age was observed in three studies [9, 17, 19]. Additionally, more male (60.4%) than female patients were included. Pooled analysis of mortality (Fig. 2) indicated no significant reduction in patients treated with vitamin C when compared to reference. Interestingly, subgroup analyses concerning timing of pooled mortality assessment (Additional file 1: Figure S1) revealed a reduction of short-term mortality (< 30 days) but no statistically significant result regarding long-term mortality in the presence of significant subgroup differences. Also, an analysis regarding the intervention’s vitamin C only versus a combination of vitamin C and thiamine or hydrocortisone was conducted indicating no statistically significant effect concerning subgroup differences (Additional file 1: Figure S2). Analysis of treatment duration indicated that treatment for 3–4 days significantly improved survival, when compared to patients treated 1–2 or > 5 days (Fig. 3). Studies with insufficient data concerning duration of therapy were excluded from this analysis [15]. In addition, analyses were conducted to assess potential biological heterogeneity (Additional file 1: Figure S3). Patients age (< 65 vs ≥ 65 years) of the studies providing mean and standard deviation was compared. Studies only providing a range/interquartile range were excluded. To provide comprehensive data on mortality, we added Additional file 1: Figure S4 with all available data on mortality within the included studies. When additionally considering length of vasopressors and ICU treatment as stated in our predefined protocol, there were only limited data as standard deviations/original data were not available to us. Therefore, after contacting the corresponding authors, we decided to omit both parameters as comprehensive data were lacking. The overall bias was judged as moderate for all included studies with a mean Jadad score of 3 (Additional file 1: Tables S3 and S4). No sign of significant publication bias was observed (Additional file 1: Figure S5).

**Table 1:** Study characteristics

Author	Inclusion criteria	Exclusion criteria	Age
Fujii	Septic shock based on Sepsis-3 consensus	Age < 18 years, do-not-resuscitate, imminent death, diagnosis of septic shock > 24 h; Contraindication/other indication for study drug	61.7 ± 15
Park	Diagnosed with septic shock during ED stay and admitted to the ICU	Age < 18 years, do-not-resuscitate	I: 69 (IQR: 60–76) C: 69 (IQR: 61–76)
Iglesias	Diagnosis of sepsis/septic shock within 12 h of admission to ICU	Age < 18 years, do-not-resuscitate or intubate, pregnant, terminal disease (e.g., stage IV cancer, end-stage heart failure), no primary diagnosis of sepsis or septic shock, immediate surgery required, HIV + CD4 < 50 mm^2^, glucose-6 phosphate dehydrogenase deficiency, transferred from another hospital, presented with sepsis or septic shock > 24 h from admission	I: 70 ± 12 C: 67 ± 14
Fowler III 2014	Diagnosis of septic shock within 48 h of admission to ICU + informed consent	NA	I: low Vit C: 30–70 I: high Vit C: 49–92 C: 54–68
Fowler III 2019	ICU admission for sepsis + acute respiratory failure, mechanical ventilation through an endotracheal tube, PaO2 to FiO2 ratio less than 300 mm Hg, bilateral opacities, new or worsening respiratory symptoms without evidence of left atrial hypertension, suspected or proven infection, and met two of four systemic inflammatory response criteria. All criteria had to be met within 24 h	Age < 18 years; allergy to vitamin C; no informed consent; more than 48 h elapsed meeting ARDS criteria; they did not have a patient surrogate or physician committed to full support; pregnant or breastfeeding; moribund and not expected to survive 24 h; required home mechanical ventilation (via tracheostomy or noninvasively); receiving home oxygen > 2 L/min; had interstitial lung disease, diffuse alveolar hemorrhage, diabetic ketoacidosis, active kidney stone	I: 54 (IQR: 39–67) C: 57 (IQR: 44–70)
Marik	Diagnosis of severe sepsis or septic shock + procalcitonin ≥ 2 ng/mL were treated with intravenous hydrocortisone, vitamin C, and thiamine	Age < 18 years, pregnant, septic patients with a PCT level < 2 ng/mL within the first 24 h of ICU admission were not treated with the vitamin C protocol	I: 58.3 ± 14.1 C: 62.2 ± 14.3
Nabil Habib	Septic shock based on Sepsis-3 consensus + at least one positive blood culture	Age < 18 years, pregnant/lactating; History of oxalate nephrolithiasis, known glucose-6 phosphate dehydrogenase deficiency, paroxysmal nocturnal hemoglobinuria, hereditary hemochromatosis, any other type of shock state or patients with mixed type of shock	I: 42.78 ± 9.49 C: 41.70 ± 10.2
Wani	Sepsis and septic shock, Sepsis-3 consensus	Age < 18 years, pregnant	I: 65 (IQR: 25–72) C: 70 (IQR: 25–72)
Litwak	International Classification of Disease (ICD)-10 code for “septic shock” + Vasopressors + adult patients	Pregnant	I: 58.3 ± 17.0 C: 60.1 ± 14.0
Chang	Sepsis and septic shock, Sepsis-3 consensus	Age < 18 years, pregnant, non-septic shock, therapy limitation	I: 59.5 ± 15.0 C: 63.7 ± 12.8
Mitchell	Sepsis or septic shock defined by the 2012/2016 Surviving Sepsis Guidelines. Patients admitted to ICU who received vitamin C, Thiamine, and Hydrocortisone	NA	I: 68 ± 10 C: 68 ± 10
Ahn	Sepsis and septic shock	Age < 18 years, pregnant, death within 24 h from ICU admission, do-not-resuscitate or intubate, and organ transplantation during ICU stay	I: 67.8 ± 12.1 C: 63.6 ± 15.3
Sadaka	Sepsis and septic shock, Sepsis-3 consensus	Age < 18 years	I: 67 ± 16 C: 70 ± 12
Zabet	Sepsis and septic shock	Age < 18 years, use of other antioxidants (e.g., vitamin E, selenium, *N*-acetylcysteine), corticosteroids, known glucose-6 phosphate dehydrogenase deficiency, and contraindication for high-dose vitamin C	I: 64.14 ± 15.98 C: 63.71 ± 13.84
Shin	Septic shock diagnosed in an ED	Age < 18 years, do-not-resuscitate order; septic shock recognized > 6 h after arrival in the ED; transferred from other hospitals/did not meet the inclusion criteria on ED arrival	I: 67 (58–76) C: 67 (60–75)
Hwang	Septic shock diagnosed in an ED, defined as sepsis with persisting hypotension, lactate levels > 2 mmol/L despite adequate fluid challenge	Age < 19 years	I: 70 (62–76) C: 69 (62–74)
Moskowitz	Sepsis and suspected/confirmed infection + vasopressors	Age < 18 years, allergic to any study drugs, kidney stones within the last year, glucose-6 phosphate dehydrogenase deficiency or hemochromatosis, kidney replacement therapy, not expected to survive 24 h	I: 68.9 ± 15 C: 67.7 ± 13.9

*HIV* human immunodeficiency virus, *ICU* intensive care unit, *NA* not available, *ED* emergency department

## Discussion

In the present systematic meta-analysis, we examined the effects of intravenous vitamin C on mortality in 17 studies including more than three thousand septic patients. Despite the lack of mortality reduction regarding the pooled mortality assessment, short-term mortality was reduced with vitamin C treatment. In addition, lower mortality rates could be demonstrated for short-term mortality and treatment duration for 3–4 days.

Sepsis represents a condition where oxidative stress prevails and ROS are insufficiently opposed by antioxidants leading to cellular injury and a dysfunctional endothelial barrier [3, 48]. Hence, low plasma concentrations of antioxidants are present in septic patients. The extent of this reduction is associated with higher mortality rates [3, 5–9, 48, 49].

Originally identified in the early twentieth century vitamin C was introduced for treatment of various diseases. However, data validated by randomized controlled studies as well as profound pathophysiological considerations were lacking in most of the cases [50]. This is different in sepsis. Sepsis represents a life-threatening condition whose pathophysiology is based on dysregulated inflammatory responses which are accompanied by low levels of antioxidants [17, 19, 49]. Sepsis is a heterogenous syndrome, and septic patients represent a heterogenous patient population. This might be one reason for the statistical heterogeneities across the studies. It is important to mention that the dosing of the administered vitamin C was quite consistent across all studies (around 6 g of vitamin C administered per day) and all treatment protocols included supraphysiologic doses which seems to be necessary to replete the extremely low plasma levels in this population [9, 52]. The duration of the intervention varied across the studies and might have impacted the results. Also, some studies used vitamin C as a monotherapy. However, sensitivity analysis regarding vitamin C only versus a combined therapy indicated no significant subgroup difference. Moreover, the inclusion of both groups improved the coverage and validity of our database concerning this intervention. Vitamin C represents an inexpensive and easily accessible drug that is considered safe even at extremely high doses (up to 710 mg/kg/day for up to 8 weeks) [9, 52]. Interestingly, six studies reported no adverse events related to the intervention [9–11, 19, 20, 24], while three studies documented more frequent adverse events in patients treated with intravenous vitamin C (hypernatremia *n* = 24, hospital-acquired infections *n* = 14, hyperglycemia *n* = 13, gastrointestinal bleeding *n* = 3, and fluid overload *n* = 1) [14, 21, 25]. The remaining eight studies did not specifically address this issue [12, 13, 15–18, 22, 23]. With regard to the large population included in this analysis, only little adverse events were reported when considering the extremely high doses of intravenous vitamin C that were used across the studies. This is in line with recent data suggesting safety and efficiency of the frequently administered combination of vitamin C, corticosteroids, and thiamine which was used in most of the included studies [2, 52]. However, it is important to consider that patients with glucose-6 phosphate dehydrogenase deficiency, paroxysmal nocturnal hemoglobinuria, hemochromatosis, nephrolithiasis, and other contraindications for vitamin C were excluded in most of the studies.

Early studies in sepsis and related conditions supported the pathophysiological rational for the use of vitamin C, with studies showing reduced mortality [11, 18], reduced vasopressors [17], and additional improved outcomes [9]. Also, the results from a recent large, randomized, controlled trial (CITRIS-ALI) showed significantly reduced mortality rates in the treatment group, and further analysis indicated reduced SOFA scores at 96 h [19, 26]. However, results from various published studies were vastly heterogenous. The HYCTSSS trial demonstrated reduced SOFA scores [14], ORANGES a shorter duration of shock [20], VITAMINS no improvement in mortality and duration of shock [21], and finally the ACTS trial revealed no improvement regarding SOFA scores and incidence of kidney failure [25]. Importantly, the authors of the ACTS trial stated that in the intervention arm, the most common reason for death was withdrawal of care due to a terminal illness, affecting 26% of the patients who died before discharge.

Against the background of eight new clinical trials published only in 2020 and the heterogeneous results, there was an urgent need for a systematic meta-analysis to assess the overall evidence. Indeed, our study may support favorable effects of vitamin C therapy in sepsis and provides novel insights for a potential optimized treatment strategy. We observed reduced mortality rates in two subgroups: treatment for 3–4 days and concerning short-time mortality (defined as no occurrence of death < 30 days of treatment in the pooled mortality assessment). However, based on the character of these analyses, the results are just hypothesis generating. Regarding treatment duration, it is important to remember that nicotinamide adenine dinucleotide phosphate oxidase and mitochondrial-generated ROS are involved in activation of lymphocytes and monocytes [53]. One could hypothesize that the use of antioxidants may be valuable during the initial exaggerated inflammatory responses but is harmful when reactive immunosuppression occurs [53, 54]. Therefore, a targeted and individual treatment strategy is desirable to promote an ideal response to infectious agents. In conclusion, patients may benefit from a rational treatment strategy for 3–4 days as presented in this analysis when compared to extremely short approaches or an excessively prolonged treatment regime [51, 53, 54]. These findings illustrate the effects of this well-tolerated intervention supporting the clinical relevance of the anti-inflammatory and antioxidant effects at least in specific subpopulations.

Previous meta-analyses focused on different endpoints such as length of ICU stay or mechanical ventilation, included diverging patient populations or a limited number of studies [31–33]. In contrast, our study homogeneously consisted of septic patients and included all studies identified with the defined search terms fulfilling the predefined quality characteristics. Furthermore, we performed various subgroup analyses generating new hypotheses for practical applications. Certainly, mortality represents an objective endpoint, which can potentially be reduced in several sepsis populations as demonstrated in the present analysis. As a result, future studies should steadily explore this endpoint and provide profound data on the included patients, follow-up, and the duration/initiation of treatment. Future studies may benefit from our findings as homogenous study designs, and consequent follow-up procedures may improve upcoming trials. In addition, future studies might focus on more subtle improvements such as length of stay or delta SOFA which were not provided by all the included studies. Another important finding deals with the magnitude of the favorable effects which were observed in the treatment group and tended to decrease over time. This is apparent when significant short-term effects are compared to nonsignificant long-term effects. Potentially, beneficial effects diminish over time or adverse events may have been delayed. Sepsis represents a complex multi-factorial disease influenced by diverse variables impacting mortality over time. Therefore, despite unfavorable results of most recently published studies, further studies are urgently needed. In this sense, the upcoming results of the VICTAS trial are of interest and may change the overall outcomes again [35].

The results are limited by the nature of the published studies, which were qualitatively assessed by the ROBINS-I criteria for observational studies and the Jadad score for randomized studies. Furthermore, dosing, treatment duration, and combination of the study drugs were partially heterogenous and might have impacted the results. Also, single-patient data on several patient characteristic, e.g., age, were lacking. Apart from the 30-day and 90-day mortality, exact timing of hospital and ICU mortality differed within the studies. Pooled analysis of mortality might lead to confounding. However, pooled mortality is of interest to potentially generate hypotheses on the overall mortality and a single endpoint for each study is necessary to provide proportionate weighting of the individual studies. Mortality as a primary outcome can be biased toward withdrawals or family decisions as this outcome can be severely impacted by and end-of-life decision [51]. In contrast, the pooling of similar patient cohorts can support the power of results. There might have been studies that were not identified by our search criteria and have been missed. The overall risk of bias of the included studies was judged as moderate. As with all meta-analyses, the risk of potential publication bias must be considered when the results are evaluated. However, no indications for relevant publication bias could be determined using Funnel plotting.

## Conclusion

In conclusion, we analyzed intravenous vitamin C therapy in sepsis summarizing the most recent available clinical data. As a result, specific subgroups of septic patients that might benefit from vitamin C were identified. Subsequent studies should focus on these subgroups. Additional aspects that need to be considered are the length of vitamin C treatment. Future studies are required to identify additional patient characteristics or verify our findings to implement a focused vitamin C treatment in septic patients.

## Supplementary Information

**Additional file 1.** Supplemental figures and tables.

## Data Availability

The datasets used and/or analyzed during the current study are available from the corresponding author on reasonable request.

## Abbreviations

ICU: Intensive care unit
RevMan: Review Manager
ROS: Reactive oxygen species
SD: Standard deviation
SOFA: Sequential Organ Failure Assessment
