# Supplementary material for: Mortality in septic patients treated with vitamin C: a systematic meta-analysis
Source: Crit Care. 2021 Jan 6;25:17. doi: 10.1186/s13054-020-03438-9 (PMC7787590; doi:10.1186/s13054-020-03438-9)
Supplement: Supplementary file 1 — Additional file 1. Supplemental figures and tables. [file 13054_2020_3438_MOESM1_ESM.docx]

**Supplement**


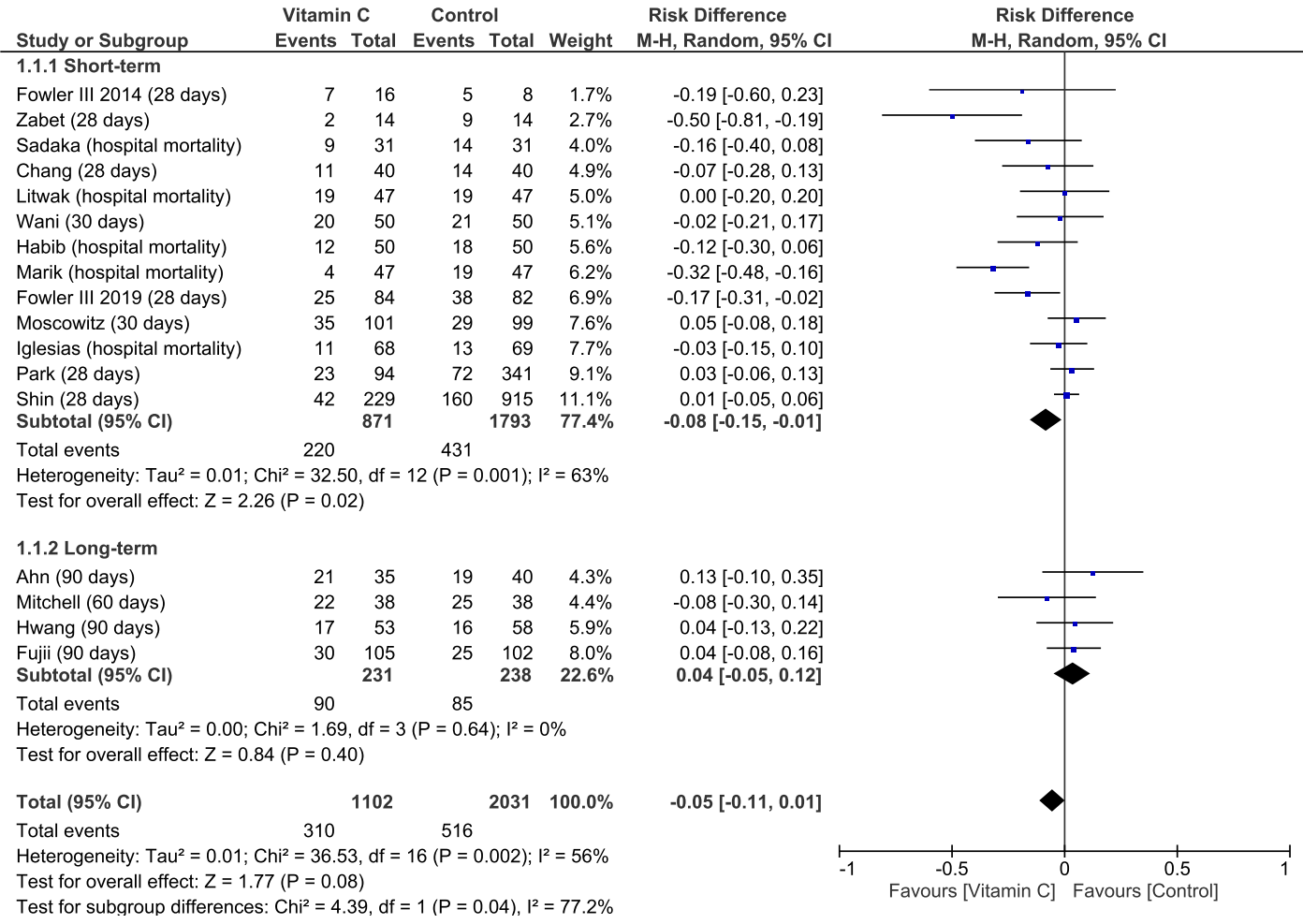


Figure S1. Pooled Mortality including subgroup analysis (date of mortality assessment), risk difference, vitamin C treatment vs. Control; M-H: Mantel-Haenszel, CI: confidence interval


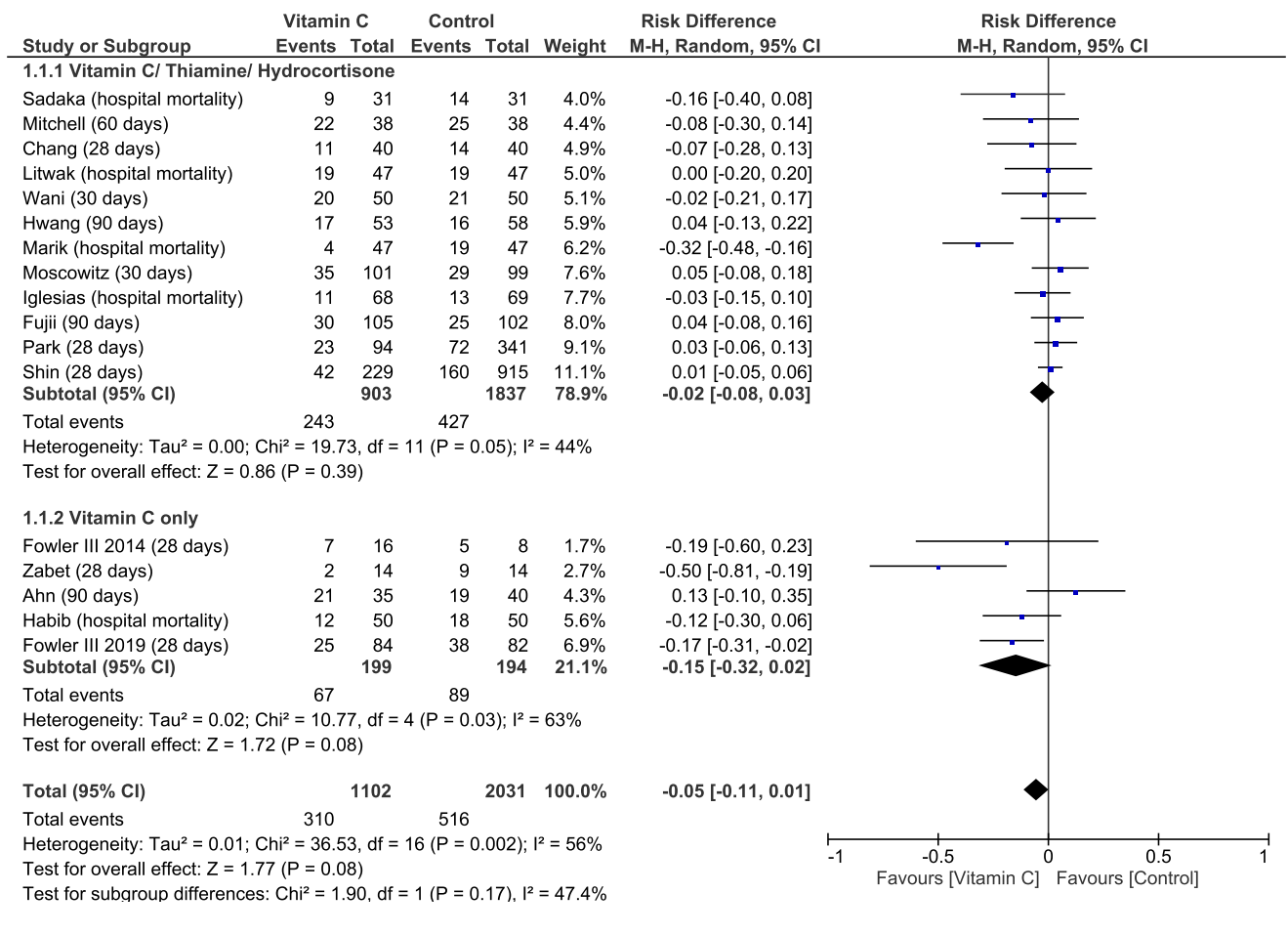


Figure S2. Pooled Mortality (date of assessment) including subgroup analysis regarding Intervention (Vitamin C only versus Combined), risk difference, vitamin C treatment vs. Control; M-H: Mantel-Haenszel, CI: confidence interval


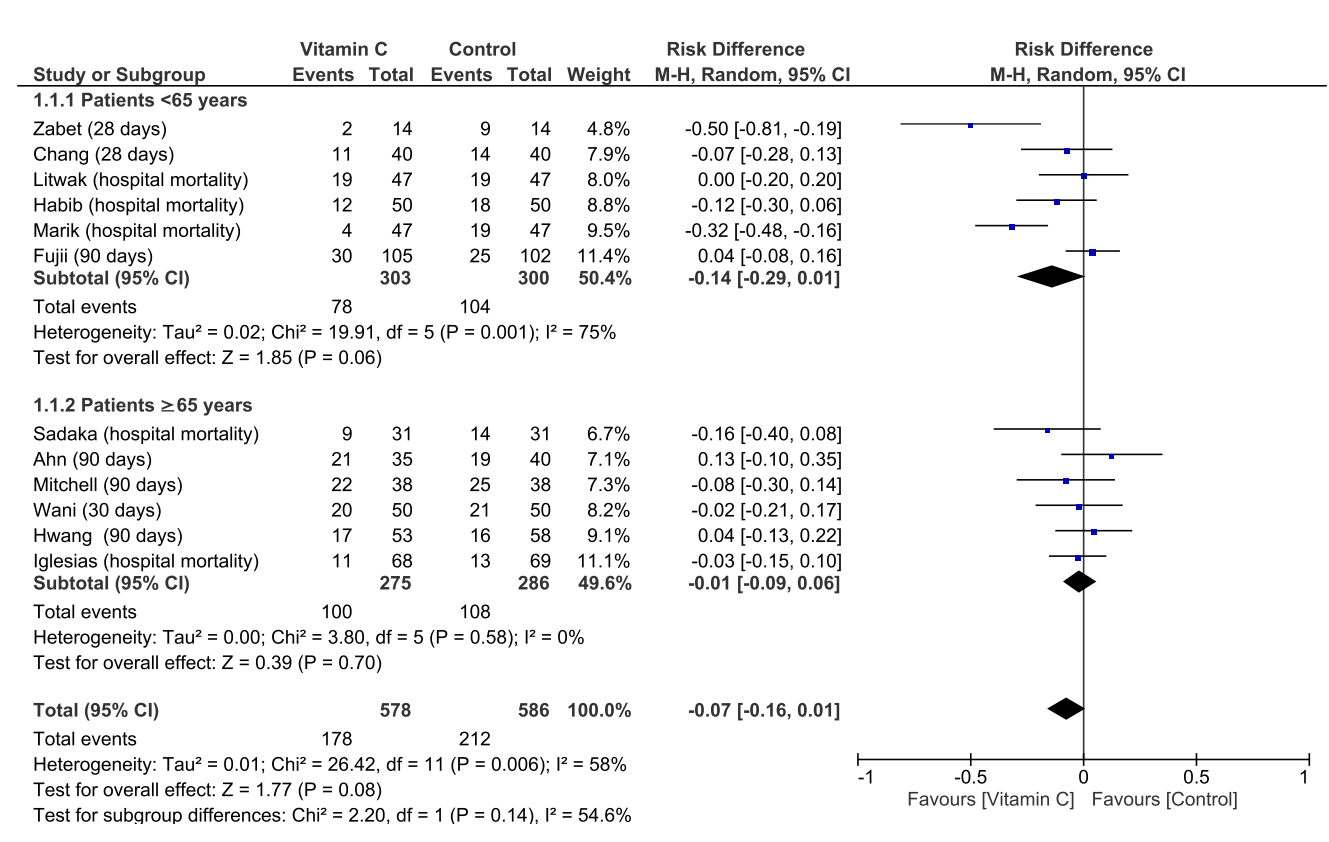


Figure S3. Pooled Mortality (date of assessment) including subgroup analysis on age, Risk difference, vitamin C treatment vs. Control; M-H: Mantel-Haenszel, CI: confidence interval


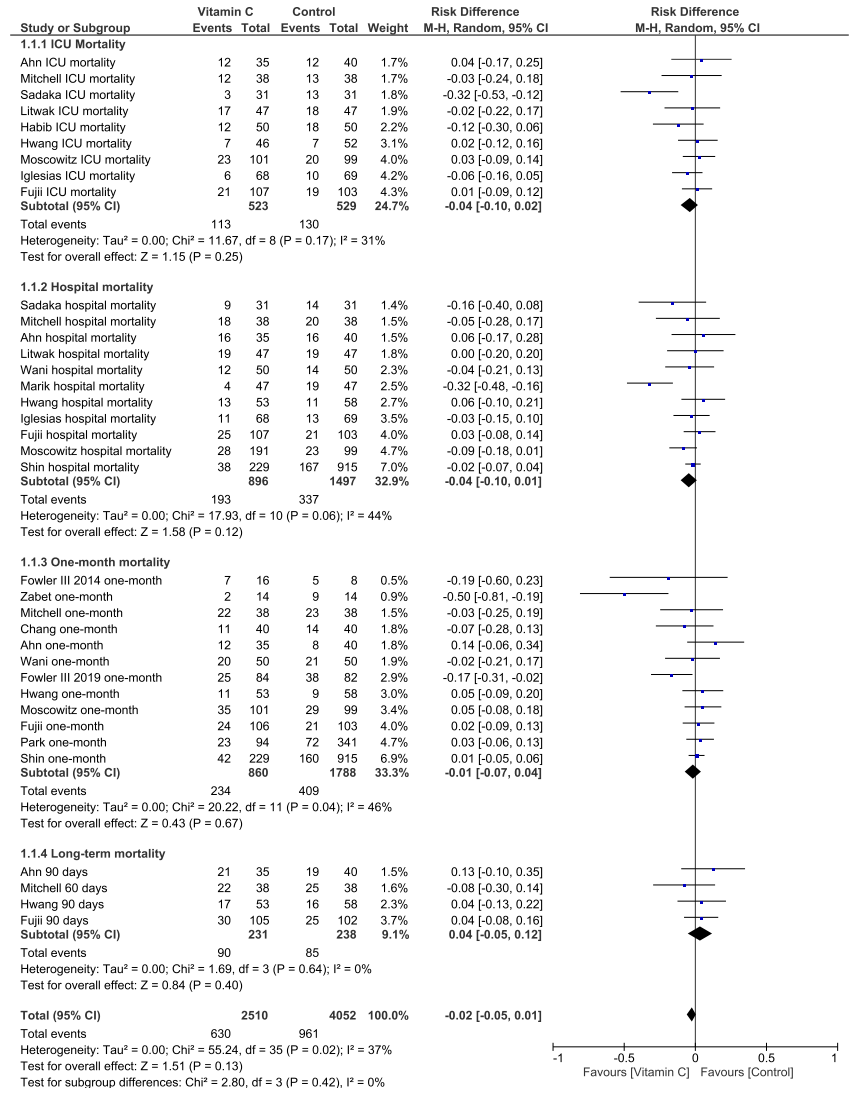


Figure S4. Mortality with all available timepoints (multiple measurements from individual studies included), Risk difference, vitamin C treatment vs. Control; M-H: Mantel-Haenszel, CI: confidence interval


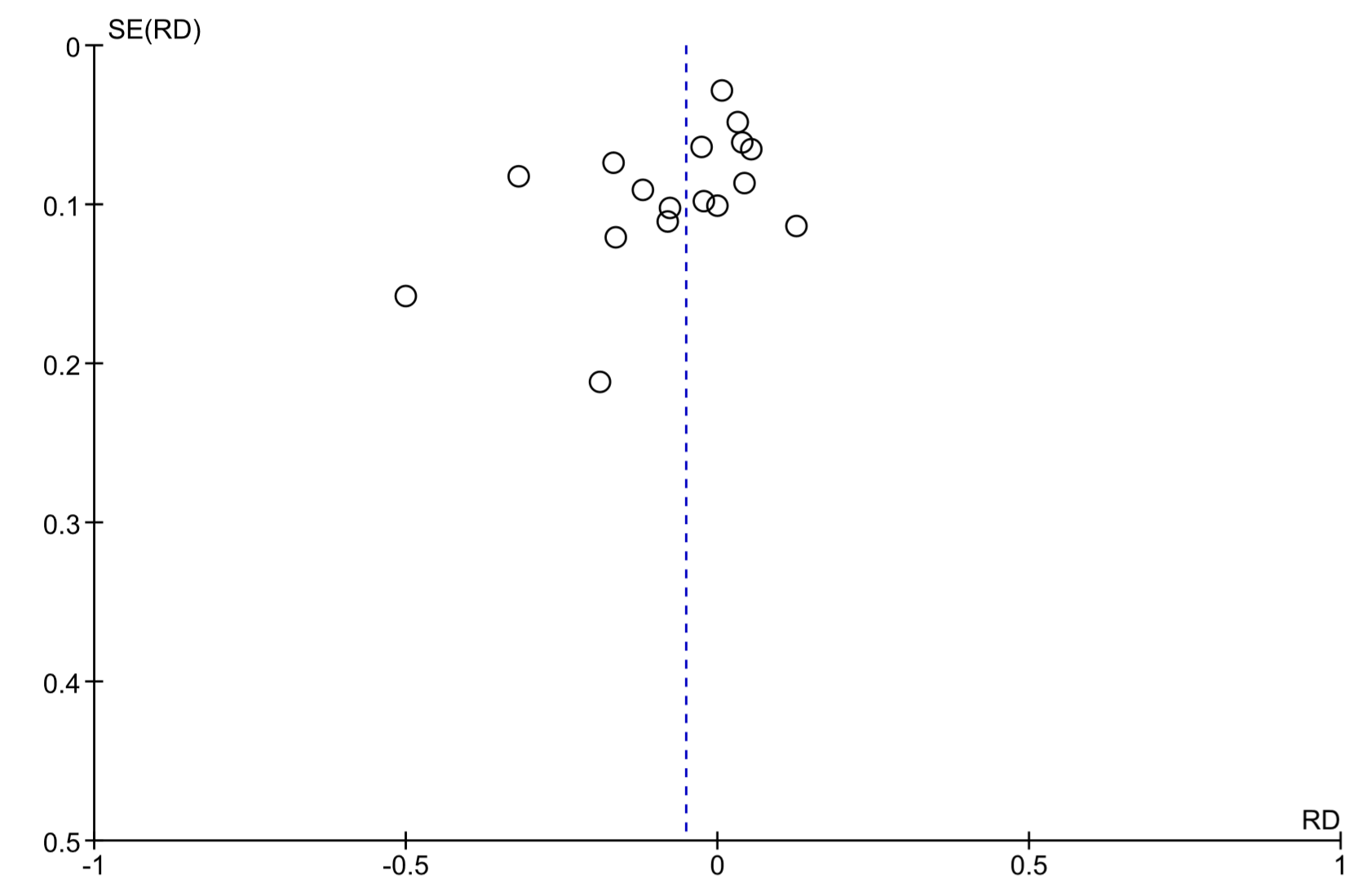


Figure S5. Funnel plot: Pooled mortality assessment; SE: Standard Error, Risk differenceTable S1. Patient characteristics

| Author  (trial) | Study design | Intervention | Comparator | 28 days mortality  (Vit C/ Control) | Longest period for data on mortality  (Vit C/ Control) |
| --- | --- | --- | --- | --- | --- |
| Fujii | Multicenter, randomized  controlled, open label | Vit C (1.5 g / 6 h), thiamine (200 mg / 12 h), and hydrocortisone (50 mg / 6 h), for 10 d; n=109 (68♂/39♀) | Hydrocortisone (50 mg/6 h), for 10 d; n=107 (65♂/39♀) | 24(106) / 21(103) | 90 d: 30(105) / 25(102) |
| Park | Single-center, retrospective | Vit C (3 g/12 h or 1.5 g / 6 h), thiamine (200 mg / 12 h) within 6 h of septic shock, median duration 2 d (IQR1-4); n=94 (55♂/39♀) | Standard treatment; n=341 (229♂/112♀) | 23(94) / 72(341) | 28 d: 23(94) / 72(341) |
| Iglesias | Two-center,  randomized, placebo controlled, double-blinded | Vit C (1.5 g / 6 h), thiamine (200 mg / 12 h), and hydrocortisone (50 mg / 6 h) for 4 d; n=68 (32♂/36♀) | Placebo for 4 d; n=69 (27♂/42♀) | NA | Hospital mortality:  11(68) / 13(69)  Hospital LOS, d  11.5±6.8 / 11±6.2 |
| Fowler III | Single-center, randomized, placebo controlled, double-blinded | Low-Vit C: 50mg kg/ 24h  high-Vit C: 200mg kg/ 24h  for 4 d; n=16 (9♂/7♀) | Placebo for 4 d  n=8 (4♂/4♀) | 3(8),4(8) / 5(8) | 28d: 3(8),4(8) / 5(8) |
| Fowler III | Multicenter,  randomized, placebo controlled, double-blinded | Vit C (50mg/kg/ 6h) for 4 d; n=84 (45♂/39♀) | Placebo for 4 d; n=82 (45♂/38♀) | 25(84) / 38(82) | 28 d: 25(84) / 38(82) |
| Marik | Single-center, retrospective, before-after study | Vit C (1.5 g / 6 h), for 4 d/ until discharge, thiamine (200 mg / 12 h) for 4 d or until discharge, and hydrocortisone (50 mg / 6 h)  for 7 d or until discharge; n=47 (27♂/20♀) | Hydrocortisone (50 mg / 6 h); n=47 (23♂/24♀) | NA | Hospital mortality:  4(47) / 19(47) |
| Nabil Habib | Single-center, randomized, controlled study | Vit C (1.5 g / 6 h), during the first 24h until ICU discharge; n= 50 (28♂/22♀)  Mean length of ICU stay, 10.0 ±5.5/ 14.1 ± 6.47 | Standard treatment; n=50 (30♂/20♀) | NA | Mortality:  12(50) / 18(50)  ICU LOS, d  10.5±5.5 / 14.1±6.5 |
| Wani | Single-center,  randomized,  controlled trial | Vit C (1.5 g / 6 h) for 4 d,  thiamine (200 mg / 12 h) for 4 d/ hydrocortisone (50 mg / 6 h) for 7 d, or until ICU discharge; n=50 (28♂/22♀) | Standard treatment; n=50 (31♂/19♀) | NA | 30 d: 20(50) / 21(50) |
| Litwak | Single-center, retrospective | Vit C (1.5 g / 6 h), 200–300 mg  thiamine (200 mg / 12 h), and hydrocortisone daily (50 mg every 6 h or 100 mg every 8 h); n=47 (28♂/19♀)  at least a single dose of the study drugs | Standard treatment; n=47 (29♂/18♀) | NA | Hospital mortality:  19(47) / 19(47)  Hospital LOS d:  IQR:19.0 (9.0-26.0)/  IQR:14.0 (8.0-23.0) |
| Chang | Single-center, single-blinded, randomized, controlled trial | Vit C (1.5 g / 6 h), for 4 d, thiamine (200 mg / 12 h) for 4 d/ or until ICU discharge, and hydrocortisone (50 mg / 6 h) for 7 d; n=40 (22♂/18♀) | Placebo treatment, n=40 (21♂/19♀) | 11(40) / 14(40) | 28 d: 11(40) / 14(40) |
| Mitchell | Single-center, retrospective | Vit C (1.5 g / 6 h), for 4 d, thiamine (200 mg / 12 h) for 4 d, and hydrocortisone (50 mg / 6 h) or (100 mg every 8 h) or continuous infusion 10 mg per kg for 7 d followed by a taper over 3-5 d; n=38 (37♂/1♀) | Hydrocortisone, n= 38 (36♂/2♀) | 22(38) / 23 (38) | 60 d: 22 (38) / 25 (38) |
| Ahn | Single-center, retrospective | Vit C (6g/d), n=35 (24♂/11♀), until ICU discharge, mean length of ICU stay >6 days | Standard treatment, n=40 (29♂/11♀) | 12(35) / 8 (40) | 90 d: 21 (35) / 19 (40) |
| Sadaka | Multicenter,  retrospective | Vit C (1.5 g / 6 h) for 4 d, thiamine (200 mg / 12 h) for 4 d, and hydrocortisone (50 mg / 6 h) for 7 d n=31 (16♂/15♀) | Standard treatment, n=31 (16♂/15♀) | NA | Hospital mortality:  9(31) / 14(31)  Hospital LOS d:  IQR:15.0 (10.0-22.0)/  IQR:9.3 (3.7-19.5) |
| Zabet | Randomized, placebo controlled double-blinded | Vit C 25 mg/kg every 6 h for 72 h, n=14 (10♂/4♀) | Placebo treatment, n=14 (11♂/3♀) | 2 (14) / 9 (14) | 28 d: 2 (14) / 9 (14) |
| Shin | Retrospective before-after | Vit C, 3 g/12 h or 1.5 g/6 h, thiamine, 200 mg/12 h) for at least 1 d, n=229 (136♂/93♀) | Reference treatment, n=915 (577♂/338♀) | 42 (229) / 160 (915) | 28 d: 42 (229) / 160 (915) |
| Hwang | Randomized Multicenter  Double-blind | Vit C (50 mg/kg, maximum single dose 3g), thiamine 200mg every 12h for 48h, n=53 (20♂/33♀) | Placebo treatment, n=58 (22♂/36♀) | 11 (53) / 9 (58) | 90 d: 17 (53) / 16 (58) |
| Moskowitz | Randomized Multicenter  Double-blind | Vit C, 1,5g/6 h, thiamine 100 mg, and hydrocortisone (50 mg/ 6 h) for 4 d n=101 (57♂/44 ♀) | Placebo treatment, n=99 (54♂/45♀) | NA | 30 d: 35 (101) / 29 (99) |

n: number of patients; ♂: male participants; ♀: female participants; d: day; h: hour, values expressed as mean ± SD or range; IQR: interquartile range; LOS: Length of stay; NA: not available

Table S2. Initiation and duration of the intervention

| Author | Initiation of therapy | Duration of therapy |
| --- | --- | --- |
| Fujii | Septic shock based on Sepsis-3 consensus had to be fulfilled 24 h prior to enrollment | Patients in the intervention group received treatment for a mean of 3.4±2.1 d |
| Park | Diagnosed with septic shock during emergency department stay and admitted to the ICU. Initiation of therapy within 6 h of shock recognition. | Median of 2 d (IQR 1-4 d) |
| Iglesias | Diagnosis of Sepsis within 12h = inclusion criterion, patients received their first dose of study treatment between 3 and 14 h (mean 9.9±4.5 h) | 3.3±0.3 d |
| Fowler III  2014 | When patients met the inclusion criteria within 48 hours they were randomized. The study drug was received after 2-4 h | Patients were infused for 4 d |
| Fowler III  2019 | All inclusion criteria had to be met within 24 h. The study drug was administered within 6 hours of randomization or at earliest available time after clinically indicated procedures. | Study drug for 96 h = 4 d |
| Marik | Within 24 h after admission | Treatment for 4 d or until ICU discharge  (median ICU LOS was 4 (3-5 d) |
| Nabil Habib | Within 24 h after admission | Until ICU Discharge, days of ICU Stay 10±5.5d |
| Wani | Triple therapy was administered within few hours of admission (in all cases <24 h) | Vit C for 4 d or until discharge from the hospital |
| Chang | Subgroup diagnosed within 48 h with favorable outcomes | Vit C for 4 d or until ICU discharge; ICU LOS 7.5 d (IQR 4-12.8) |
| Mitchell | Patients admitted to ICU who received vitamin C, Thiamine, and Hydrocortisone (retrospective) | Vit C for 4 d or until discharge |
| Ahn | Therapy was started on the first working day after ICU admission regardless of the severity of a patient | Vit C admission until ICU discharge; ICU LOS 10d (6-19d) |
| Sadaka | Sepsis and septic shock, Sepsis-3 consensus (retrospective) | Patients were included if the received Vit C for full 4 d |
| Zabet | NA | For 72 h = 3 d |
| Shin | Within 6 h of shock recognition. | 1 d; further admission based on physician’s preference |
| Hwang | Septic Shock diagnosed in an ED meeting the inclusion criteria within 24 h after ED arrival | 2 d |
| Moskowitz | Sepsis and suspected/ confirmed infection + Vasopressors. Patients were enrolled within 24 h once they were identified. | Vit C for 4 d or until ICU discharge |

d: day; h: hour, ED: emergency department, values expressed as mean ± SD or range; IQR: interquartile range; LOS: Length of stay

Table S3. Risk of Bias in Non-Randomized Studies (ROBINS-I)

|  | **Confounding** | **Selection of participants** | **Classification** | **Deviations from interventions** | **Missing data** | **Measurement of Outcomes** | **Selective reporting** | **Judgement of overall Bias** |
| --- | --- | --- | --- | --- | --- | --- | --- | --- |
| **Sadaka** | U | U | U | N | PN | PN | PN | **moderate** |
| **Ahn** | PY | PY | PN | N | PN | PN | PN | **moderate** |
| **Mitchell** | U | U | PN | N | PN | PN | PN | **moderate** |
| **Litwak** | U | U | U | N | PN | PN | PN | **moderate** |
| **Marik** | PN | PN | PN | PY | PN | PN | PN | **moderate** |
| **Park** | U | U | U | PY | PN | PN | PN | **moderate** |
| **Shin** | PN | U | PN | N | PY | U | PN | **moderate** |

PY: probably high; PN: probably low; Y: high; N: low; U: unclear

Table S4. Jadad Score

|  | **Randomization** | **Blinding** | **Lost to follow-up** | **Total** |
| --- | --- | --- | --- | --- |
| **Fowler 2014** | 2 | 2 | 0 | **4** |
| **Zabet** | 2 | 2 | 0 | **4** |
| **Chang** | 1 | 0 | 1 | **2** |
| **Wani** | 1 | 0 | 0 | **1** |
| **Habib** | 2 | 0 | 0 | **2** |
| **Fowler 2019** | 2 | 2 | 1 | **5** |
| **Iglesias** | 2 | 1 | 0 | **3** |
| **Fujii** | 2 | 0 | 1 | **3** |
| **Hwang** | 2 | 2 | 1 | **5** |
| **Moscowitz** | 2 | 2 | 1 | **5** |
